# How to scale-up: a comparative case study of scaling up a district health management strengthening intervention in Ghana, Malawi and Uganda

**DOI:** 10.1186/s12913-023-09034-1

**Published:** 2023-01-16

**Authors:** Susan Bulthuis, Maryse Kok, Olivier Onvlee, Thomasena O’Byrne, Samuel Amon, Justine Namakula, Kingsley Chikaphupha, Jana Gerold, Wesam Mansour, Joanna Raven, Jacqueline E. W. Broerse, Marjolein Dieleman

**Affiliations:** 1 KIT Royal Tropical Institute, Amsterdam, The Netherlands; 2 Athena Institute, VU University, Amsterdam, the Netherlands; 3 Trinity College Dublin, Dublin, Ireland; 4 Department of Health Policy, Planning & Management, School of Public Health, College of Health Sciences, University of Ghana, Accra, Legon Ghana; 5 Makerere University School of Public Health, Kampala, Uganda; 6 Research for Equity and Community Health (REACH) Trust, Lilongwe, Malawi; 7 Swiss Centre for International Health, Swiss Tropical and Public Health Institute, University of Basel, Allschwil, Switzerland; 8 Department of International Public Health, Liverpool School of Tropical Medicine, Liverpool, UK

**Keywords:** Scale-up, Barriers and facilitators, District health management strengthening intervention, LMICs, Comparative case-study

## Abstract

**Background:**

The need to scale up public health interventions in low- and middle-income countries to ensure equitable and sustainable impact is widely acknowledged. However, there has been little understanding of how projects have sought to address the importance of scale-up in the design and implementation of their initiatives. This paper aims to gain insight into the facilitators of the scale-up of a district-level health management strengthening intervention in Ghana, Malawi and Uganda.

**Methods:**

The study took a comparative case study approach with two rounds of data collection (2019 and 2021) in which a combination of different qualitative methods was applied. Interviews and group discussions took place with district, regional and national stakeholders who were involved in the implementation and scale-up of the intervention.

**Results:**

A shared vision among the different stakeholders about how to institutionalize the intervention into the existing system facilitated scale-up. The importance of champions was also identified, as they influence buy-in from key decision makers, and when decision makers are convinced, political and financial support for scale-up can increase. In two countries, a specific window of opportunity facilitated scale-up. Taking a flexible approach towards scale-up, allowing adaptations of the intervention and the scale-up strategy to the context, was also identified as a facilitator. The context of decentralization and the politics and power relations between stakeholders involved also influenced scale-up.

**Conclusions:**

Despite the identification of the facilitators of the scale-up, full integration of the intervention into the health system has proven challenging in all countries. Approaching scale-up from a systems change perspective could be useful in future scale-up efforts, as it focuses on sustainable systems change at scale (e.g. improving district health management) by testing a combination of interventions that could contribute to the envisaged change, rather than horizontally scaling up and trying to embed one particular intervention in the system.

**Supplementary Information:**

The online version contains supplementary material available at 10.1186/s12913-023-09034-1.

## Background

The need to scale up public health interventions in low- and middle-income countries to ensure equitable and sustainable impact is widely acknowledged [1–3]. Less straightforward is, however, how to scale up public health interventions, as there is no universally replicable approach. Scale-up can be defined as “deliberate efforts to increase the impact of successfully tested pilot, demonstration or experimental projects to benefit more people and to foster policy and program development on a lasting basis” [4]. In particular, vertical scale-up – the institutionalization of a public health intervention in the national health system – is complex, as it is a non-linear process, involves a variety of stakeholders and takes place in complex health systems [5]. Because of the limited understanding of how to deal with this complexity, only a few successfully tested interventions are scaled up and become part of national systems [5, 6].

Over the last 20 years, there has been increased interest in scale-up to achieve Universal Health Coverage [7]. This has led to more tangible experiences with scale-up practice, research and critical reflection, which has contributed to a ‘new understanding’ of good practices around scale-up. For example, policymakers and implementers increasingly consider and reflect on how to scale up from the beginning of implementation of an intervention, instead of scale-up being an ‘afterthought’ [6, 8]. Other notable changes in approaching scale-up are considering the political economy during scale-up and taking a holistic systems approach. There are tools and guidelines available that have emerged based on this improved understanding [7]. However, as Ghiron, Ramirez-Ferrero [6] recently stated, “there has been little documentation of how large multi-country, donor-funded projects have sought to address the importance of scale-up in the design and implementation of their initiatives”. The aim of this study is to address this knowledge gap and to provide insight in which factors and processes, from now onwards called “facilitators”, positively facilitated the scale-up of a specific health intervention – the district-level health management strengthening intervention – in contexts which are shaped by different systemic and general contextual factors. This study was conducted within the framework of the PERFORM2Scale project, which sought to scale up a health management strengthening intervention targeting district health managers in Ghana, Malawi and Uganda over a five-year period (2017–2021) to contribute to Universal Health Coverage.

### PERFORM2Scale – intervention and scale-up framework

The management strengthening intervention was developed by the PERFORM project and piloted together with district health management teams (DHMTs) in nine districts in three sub-Saharan African countries between 2011 and 2015. Positive outcomes, such as strengthened management competencies to improve health workforce performance and service delivery, and a high level of acceptability of the intervention by the district health managers [9] led the European Union (the funder) to decide, with support from the Ministries of Health in Ghana, Malawi and Uganda, to fund a project to scale up the intervention (2017–2021).

The intervention aimed to reinforce the management capacities of DMHTs to expand the use of their decision space. Country research teams^1^ helped DHMTs to go through different steps of an action research cycle: plan, act, observe and reflect. In the first step (‘plan’), the DHMT members conducted – through various workshops – a situation analysis, identified a problem, analysed root causes and developed a work plan to address the problem. In the second step (‘act’), the DHMTs implemented their work plans with their own financial and human resources available, over a period of 8–12 months, in line with the existing district planning cycles. Observation (step 3) took place through monitoring the effects of interventions implemented as part of the work plans using indicators as depicted in the work plans. Through follow-up visits by country teams and inter-district meetings (where districts shared their experiences with implementation of the intervention), observation and reflection (steps 3 and 4) were encouraged. To facilitate reflection, DHMTs used diaries to document and assess the implementation of their action plans. Based on these reflections, DHMTs could decide to adapt the activities and work plan to address the problem, continue with the same work plan, or select another problem for another action research cycle.^2^

The PERFORM2Scale consortium adapted a scale-up framework – the ExpandNet framework [4] – that has been developed to guide “the strategic planning and management of the scaling up process” (p.7). The framework distinguishes five key interacting elements: (i) the innovation; (ii) the user organization; (iii) the resource team; (iv) the environment; and (v) the scale-up strategy. In this last element, the different elements of the framework come together, as the scale-up strategy should contain strategic choices concerning dissemination and advocacy, organizational processes, cost and resource mobilization, and monitoring and evaluation. In the context of PERFORM2Scale, the innovation is the management strengthening intervention, which proved to be a scalable intervention based on an assessment of the scalability of the intervention [10]. The user organization in Uganda is the Ministry of Health (MoH), in Malawi it is the MoH and the Ministry of Local Government, and in Ghana it is the MoH and the Ghana Health Service. Representatives of these ministries are members of the National Scale-up Steering Group, and oversee and steer the scale-up. It was envisaged that the country teams would implement the intervention in the first districts and that this would gradually become the responsibility of the resource team. Depending on the country, resource team members were district, regional and national management-level staff from the abovementioned ministries. The environment is the context in which the scale-up takes place, which was assessed in three country specific initial context analyses in 2017 [11].

A central element of the framework, the scale-up strategy, focuses on the actions to be taken to ensure that the intervention, resource team and steering group have the right attributes for successful scale-up in a specific environment. The development of a scale-up strategy enables strategic choices depending on the types of scale-up envisaged. PERFORM2Scale aimed for horizontal scale-up (the spread of the intervention to an increasing number of districts) and vertical scale-up (the institutionalization of the intervention in policies, budgets and strategies). PERFORM2Scale developed a generic strategy for horizontal scale-up. This strategy described that in each country, the implementation of the intervention would start in three districts (district group 1 in 2018) for a first action cycle of eight months, after which this would be scaled up to three other districts (district group 2 in 2019), followed again by three other districts (district group 3 in 2020). The idea of the resource team was that it would ultimately take over the role of the country teams in facilitating the intervention and in expanding the number of districts. Furthermore, it was planned that the project would finance the implementation in the first nine districts (this included the facilitation of the workshops, but not the activities of the DHMTs identified in the work plans – they were financed from existing district resources), and that through continuous engagements with the steering groups (and resource teams), a country-specific strategy for vertical scale-up would be developed in the project period to ensure that the intervention would be implemented beyond the nine districts funded by the project. Table 1 provides an overview of the scale-up structures and approaches in the three countries.

**Table 1:** Overview of country-specific scale-up structures and approaches (scale-up strategies)

	**Ghana**	**Malawi**	**Uganda**
Steering Group (user organization)	The steering group consists of national-level stakeholders from the Ghana Health Service, the MoH and the Christian Health Association of Ghana. It also includes the Regional Director of the region where the intervention is being implemented	The steering group consists of four directors of relevant MoH departments (Quality Management, Human Resources Management and Development, Planning and Policy Development and Clinical Medicine), one director from the Ministry of Local Government and one from the Department of Human Resource Management and Development in the Office of the President and the Cabinet	Steering group members were mostly commissioners of relevant departments of the MoH (Human Resource Management, Health Services (Planning) and Quality Assurance and Improvement) and included the Director-General. However, the steering group did not take off as a group. Therefore, two adaptations were made: 1) a steering group focal person was appointed, who could regularly update the other members; and 2) present to technical working groups at the MoH, to which some of the steering group members belonged
Resource team	Six members (varied over time). The resource team comprises DHMT members who have been involved in the intervention, and two regional actors (including the Regional Director), of which one is the chair. When district numbers increased, the number of resource team members also increased	Eight members. Three members are deputy directors from relevant departments of the MoH and the Ministry of Local Government. Three members are MoH officers at zonal/satellite level, and one is a District Director of Health and Social Services. One team member is from the staff development institute	Four members, drawn from the departments to which the previous steering group members belong (mentioned above). They are mainly assistant commissioners and principal officers
Horizontal scale-up approach	The horizontal scale-up took place in districts of one specific region. The districts were selected based on the social development and service delivery performance ranking and geographical location. Selection of districts was discussed by the country team and the resource team and confirmed by the steering group	The districts were selected based on a minimal number of development partners working in health system strengthening, so that the impact of the intervention could be better measured. The district groups were selected by the country team, resource team and steering group	The intervention took place in nine districts, equally distributed across three regions. The district selection was guided by the need to facilitate peer-to-peer/cross-district learning within a region. Hence, one of the districts in each district group had been involved in the pilot phase of the intervention, and two other, new districts share borders with that district. The district groups were selected by the country team, resource team and steering group
Vertical scale-up approach	No clear entry point for integration or institutionalization of the intervention has been identified (yet)	The vertical scale-up of the intervention is envisaged by integrating the intervention in the activities of the zonal/satellite offices (responsible for technical advice to DHMTs), which are part of the Quality Management Department of the MoH. The intervention workshops are envisaged to be integrated into their quarterly review meetings, and satellite officers will take over the facilitating roles of the country team and resource team. Furthermore, elements of the intervention have been integrated into the national integrated supportive supervision tool	The vision is that certain components of the intervention will be integrated into the revised version of the MoH’s quality improvement strategy for 2021–2025

Table 1 shows that the scale-up structures have been functioning differently and that the horizontal and vertical scale-up approaches differed across the countries. This paper analyses the scale-up of the intervention in the different countries over time and explores the facilitators of the scale-up in Ghana, Malawi and Uganda.

## Methods

A comparative case study approach was taken to identify the facilitators in the scale-up across three different settings in sub-Saharan Africa: Ghana, Malawi and Uganda. This approach allows identification of similarities, differences and patterns across different cases [12]. The three countries were selected by PERFORM2Scale based on differences in the type and length of their decentralization process. In Supplementary file 1, a figure is provided illustrating the different types of decentralized health systems in Ghana, Malawi and Uganda.

### Study process and data collection

A combination of different qualitative methods was used. Data collection took place in two rounds (April–June 2019 and April–May 2021).

#### *In-depth *interviews

During the first round of data collection, in-depth interviews with DHMT members took place to gain insights into their experiences with the intervention and the horizontal scale-up. In the first round of data collection, a pilot-tested interview guide was used, which included reflective questions about the different steps of the intervention. Interviews were held with three participants per district per country in the three districts (district group 1) where implementation of the intervention had taken place.

For the second round, the interview guide was adapted slightly, and more attention was paid to validation of the processes (using existing documentation on what happened in the districts) and gaining a deeper understanding of factors that influenced the horizontal scale-up. Interviews were held in four districts (two districts from district group 1 and two districts from district group 2) in Ghana and Malawi, and all six districts from districts groups 1 and 2 in Uganda (see Table 2). In general, interviews took 60–90 min. Participants were purposefully sampled based on their engagement with the horizontal scale-up. Selection of DHMT members took into account their function, type and level of involvement, gender and seniority. In Uganda, a human resource officer of the district council was included, because this office was involved in the intervention, which was not the case in the other countries.

**Table 2:** Overview of participants

		First round (April–June 2019)	Second round (April–May 2021)
In-depth interviews	Ghana	3 districts (DG1^*^): 9 total - 9 DHMT members	4 districts (DG1 and DG2): 11 total - 11 DHMT members
	Malawi	3 districts (DG1): 9 total - 9 DHMT members	4 districts (DG1 and DG2): 12 total - 11 DHMT members - 1 district council
	Uganda	3 districts (DG1): 10 total - 9 DHMT members - 1 Human Resources Officer of district council	6 districts (DG1 and DG2): 16 total - 15 DHMT members - 1 Human Resources Officer
Scale-up assessment	Ghana	- 3 steering group members (group discussion) - 3 resource team members (group discussion)	- 3 steering group members (individual) - 5 resource team members (group discussion)
	Malawi	- 1 steering group member (individual) - 2 resource team members (individual)	- 2 steering group members (individual) - 5 resource team members (group discussion) - Additional stakeholders: 1 government, 1 United Nations organization and 1 bilateral donor
	Uganda	- 3 resource team members (individual)	- 1 resource team member - 1 steering group member
Country team reflection	Ghana	- 4 country team members (group discussion)	- 4 country team members (group discussion)
	Malawi	- 4 country team members (group discussion)	- 4 country team members (group discussion)
	Uganda	N.A	- 3 country team members (group discussion)

^*^DG district group

#### Scale-up assessment

Second, a scale-up assessment was conducted, during which the resource team and steering group members considered the scale-up of the intervention and influencing factors. The members individually scored statements about the scale-up, and then a group discussion took place about their scores. The statements were informed by a literature review that identified barriers and facilitators to scale-up of public health interventions [13]. An interview guide was developed for the group discussions. During the first round of data collection, the scale-up assessment in Ghana entailed two group interviews, one with three steering group members and one with three resource team members. In Uganda and Malawi, the scale-up assessments were conducted as individual interviews, as it was impossible to bring participants together due to their busy schedules.

For the second round of data collection, the scale-up assessment with the resource team in Ghana and Malawi took place as group discussions. With all others (the steering group members in Ghana, Malawi and Uganda, and the resource team in Uganda), individual interviews were conducted. In Malawi, round 2 interviews also took place with additional stakeholders, as, in addition to the resource team and steering group, more stakeholders were knowledgeable about the scale-up of the intervention. The interviews and group discussions took about 90 min.

#### Reflective sessions

Reflective sessions with the country teams took place. During those group discussions, facilitators from KIT Royal Tropical Institute supported the country team members to reflect on the implementation of the intervention and its scale-up and influencing factors. The facilitators used an interview guide, which was based on the scale-up assessment interview guide. These sessions facilitated deeper reflection of the findings from the in-depth interviews and scale-up assessment. During the first round of data collection, the country team reflection took place in Ghana and Malawi. The reflective session did not take place in Uganda because of logistic reasons. During the second round of data collection, the country team reflection took place in all three countries. The reflective sessions took about 90 min. Table 2 provides an overview of the participants during the two rounds of data collection.

During the first round of data collection (2019), interviews and group discussions took place face to face. Because of COVID-19, the second round of data collection (2021) took place online, except in Malawi, where COVID-19 restrictions were less stringent. Researchers from KIT Royal Tropical Institute (authors SB, MK, OO) and Trinity College (author TO’B) conducted all data collection; they had not been involved in the implementation and scale-up of the intervention. In the first round of data collection in Ghana, research assistants supported the data collection. The consortium extensively discussed all guides, to adapt them to each country context.

### Analysis

The interviews and group discussions were recorded and transcribed verbatim. As a way of structuring the data, a deductive coding approach, based on the topic guides, was conducted by authors SB and OO. In addition, an inductive approach was taken to identify emerging themes or sub-themes concerning the facilitators of the scale-up in the three countries. Descriptions of main findings for each theme were presented in a matrix for each country, which were discussed and validated by all co-authors. After this, narratives were developed on the scale-up in each country and on the facilitators of the scale-up across the countries. The analysis was supported by NVivo software for qualitative data analysis.

### Ethics

The Research Ethics Committee of the authors' institutes provided ethical approval. Interviews and discussions were conducted in private, and informed consent processes were followed, with written consent being provided. Permission was sought before recording the interviews and discussions. Data were managed, stored, analysed and presented ensuring full confidentiality.

## Results

We first present details on the vertical and horizontal scale-up in each country, after which the facilitators are presented.

### Scale-up

#### Ghana

The horizontal scale-up in Ghana advanced well, and over the course of PERFORM2Scale the resource team members assumed responsibility and mostly took the lead in the facilitation of the intervention workshops and monitoring visits. The composition of the resource team, mainly DHMT members from the districts involved, resulted in sufficient availability and engagement. During the horizontal scale-up, the intervention was not substantially adapted.

The vertical scale-up in Ghana was challenging, and lacked a strategy. The country team and steering group participants discussed that the steering group had changed from an active body, with regular meetings at the beginning of PERFORM2Scale (2018), to an inactive body without meetings towards the end (2021). In 2020 and 2021, one steering group member was transferred, and two retired. Furthermore, steering group and country team participants pointed out that the steering group members are high-level officials and, therefore, extremely busy. In the absence of a vertical scale-up strategy and accompanying budget, it remains to be seen whether the resource team is a sufficiently sustainable structure for the continuation of the horizontal scale-up.

#### Uganda

In Uganda, the country team guided implementation of the horizontal scale-up. A steering group member and a resource team member indicated that resource team members had the right expertise for the function, but that due to their senior positions, time restrictions prevented them from visiting the DHMTs regularly. Furthermore, as PERFORM2Scale progressed, some changes in the resource team occurred due to transfers and a death. The resource team did not take over the lead role from the country team in terms of facilitating the implementation of the intervention, but it did provide policy guidance and direction to the DHMTs when necessary. The resource team suggested, therefore, using existing structures for the facilitation of the horizontal scale-up instead, such as regional quality improvement (QI) teams or departments at the regional referral hospitals.

Concerning the vertical scale-up, participants in Uganda explained that busy schedules prevented the intended steering group members from participating in regular meetings. However, in close collaboration with the steering group focal person, certain components of the intervention have been proposed for integration in the MoH’s QI strategy. The QI strategy is a national guiding document with the goal to ensure that all people in Uganda have access to quality health care services. It describes priority interventions under six strategic objectives, which are supposed to be implemented through the application of an iterative cycle of improvements (the Plan, Do, Study, Act – PDSA – cycle). The country team and steering group focal person have been lobbying for the inclusion of reflection and human resources/workforce performance elements from the PERFORM2Scale intervention in the draft of the new QI framework (2020/21–2024/25). The approval process for the improved QI framework took time, given that it had to be presented to, and approved by, various MoH committees: first, the technical working groups, then the senior management committee, eventually ending with approval from the MoH’s top management committee. This process was still ongoing due to COVID-19 delays at the time of data collection, but it has now been finalized, and elements are being integrated into the Quality Improvement Strategic Plan and Framework 2021–2025 [14].

#### Malawi

From 2020, the horizontal scale-up was led in Malawi by the resource team and supported by the country team. Resource team and country team participants explained that DHMT members have not yet internalized the intervention approach sufficiently to ensure its routine implementation without support. This became apparent during the second round of data collection, when many DHMTs admitted that the use of the management strengthening intervention became inactive, partly due to the COVID-19 pandemic. Besides steering the horizontal scale-up, resource team members were involved in the more strategic discussion related to vertical scale-up at central level. Participants stated that if the role of the resource team is taken over by the satellite offices (as envisaged for vertical scale-up), the resource team members could be seen as a pool of experts that can advise on the horizontal scale-up/intervention implementation when needed.

In Malawi, participants from the resource team, steering group and country team explained that although the steering group contains the right people, its functionality is suboptimal, and the members are not actively steering the vertical scale-up. The functioning of the steering group was influenced by busy agendas, but also inter- and intra-departmental power dynamics that resulted in limited engagement and collaboration. The country team, resource team and the steering group chair (from the Quality Management Department of the MoH) identified plans for the vertical scale-up of the intervention at the beginning of 2021. This entailed integration of the intervention in the activities of the zonal/satellite offices (responsible for technical advice to DHMTs), which are part of the Quality Management Department of the MoH. The intervention workshops are envisaged to be integrated into their quarterly review meetings, and satellite officers would take over the facilitating roles of the country team and resource team (including providing support to DHMTs during their action research cycles). In addition, in 2021, elements of the intervention (in particular a context analysis tool used by DHMTs in the plan step of the action research cycle) were integrated into the national integrated supportive supervision tool. This tool is to be used by DHMTs to assess and improve service delivery in their district.

### Scale-up facilitators

A multitude of scale-up facilitators have been identified in the three countries. Table 3 provides an overview of the main emerging facilitators and their presence in the different countries, based on the qualitative study, which is a representation of what the study participants have brought forward.

**Table 3:** Overview of facilitators of the scale-up of the management strengthening intervention

Scale-up facilitators	Ghana	Uganda	Malawi
Shared vision	-	+	+
Change agents (champions)	±	+	+
Buy-in from key decision makers	-	-	±
Political and financial support	-	-	±
Window of opportunity	-	+	+
Flexible approach	±	+	+
Understanding and using the context of decentralization	-	x	+

+  = present,—= absent, ±  = not fully present nor fully absent, X = not mentioned by participants

#### Shared vision

In Malawi and Uganda, several participants at national level explained that a clear shared vision among the different stakeholders involved of how to institutionalize (components of) the intervention into existing systems facilitated the scale-up. Country team participants in Malawi, however, acknowledged that the collaborative approach that was taken towards the shared vision could have been better. They mentioned that strong collaboration with the Quality Management Department had been key in identifying opportunities for integration of the intervention into existing structures, but also resulted in limited input from other stakeholders in developing the content of the strategy for vertical scale-up.

In Ghana, according to participants at different levels, the absence of a specific strategy on how and where to integrate the intervention into existing structures formed a barrier to scale-up. Interviewees from the steering group and resource team in Ghana indicated that the absence of a clear vision was related to confusion among stakeholders on what scale-up means and what PERFORM2Scale aimed to do concerning scale-up. Country team participants explained that during the first two years of PERFORM2Scale, the focus was on the implementation and horizontal scale-up of the intervention, and less attention was paid to the vertical scale-up. Steering group and resource team participants confirmed this and explained that they thought that the horizontal scale-up of the intervention in the Eastern Region was seen as a pilot to create evidence supporting the need for vertical scale-up.

“We have all agreed that we have gathered enough evidence to support the scale-up of the intervention in other districts, but then we still have a few more steps to go ..., to talk to the major stakeholders involved with the scaling up of the intervention.” —Resource team member, Ghana, 2021

#### Change agents

In both Uganda and Malawi, the presence and engagement of a few change agents advocating for and championing the vertical scale-up of the intervention was identified as a key facilitating factor of scale-up. In Uganda, a high number of participants, at all different levels, highlighted that the steering group focal person became a strong champion of the intervention and guided the country team in the different steps to take for vertical scale-up to happen. Although the resource team played a crucial role in the horizontal scale-up in Uganda, after the decision to integrate aspects of the intervention into the QI framework, one member became a change agent, because he was leading the QI framework validation process and could, therefore, accelerate the integration of the intervention into the QI framework. In Malawi, several participants at district and national level identified resource team members and the Director of the Quality Management Department as champions for the scale-up of the intervention. Because of the department’s specific QI mandate, they have been able to create interest in scaling up the intervention at the highest level (MoH and senior management team). In Ghana, participants at district and national level explained that champions were mostly present at district level and a few at regional level, but they were unable to influence national-level decision makers. At national level, three active champions emerged but retired or were transferred.

#### Buy-in from key decision makers

Across the three countries, it was noted that buy-in from key decision makers is critical for scale-up to happen. However, getting key decision makers involved was experienced as challenging. The initial idea behind setting up the steering group was to facilitate involvement of key decision makers, but in all three countries the steering group had limited functionality, mostly due to busy schedules. In Ghana, several participants from the steering group and country team explained that the suboptimal functionality of the steering group resulted in missed opportunities for dissemination and advocacy, and a lack of engagement of key stakeholders at national level. The majority of national-level stakeholders were unaware of the intervention and its potential scale-up. In Uganda, several participants from national level and the country team mentioned that the steering group did not function, but it included one main contact person (the change agent described above) at the MoH for vertical scale-up of the intervention. This change agent guided the country team on which steps to take to involve key decision makers: presenting to and convincing the relevant technical working groups in the MoH. However, the country team stated that they experienced resistance from key decision makers in the technical working groups regarding the scale-up of the intervention. According to the country team and steering group participants, the major cause of resistance was that the action research cycle used in the intervention was perceived as being very similar to the PDSA cycle already being used in the QI framework and strategy. Other areas of concern mentioned during the scale-up assessment and country team reflection, were related to the use of mostly qualitative data as evidence about the benefits of the intervention, and the absence of continuous financial support from PERFORM2Scale for the whole scale-up (which goes beyond the project time line). In Malawi, despite the difficulties with the functionality of the steering group, participants from the steering group, resource team and country team mentioned that there has been strong collaboration between the country team and the Quality Management Department. The Quality Management Department includes key decision makers concerning the scale-up of the intervention, and this has the potential to facilitate the scale-up. However, intra-departmental power dynamics resulted in limited engagement and collaboration with key decision makers from other MoH departments, such as the Planning Department.

#### Political and financial support

Strongly related to the endorsement by key decision makers was the (lack of) political and financial support for the scale-up of the intervention. In Uganda, the country team and steering group focal person said they were generally positive about the chances that components of the intervention will be integrated into the existing QI framework, but creating broader political support remains crucial for the vertical scale-up to actually succeed. Engaging certain commissioners, such as the Commissioner for Quality Improvement and Human Resources Development, and the senior management committee was, therefore, identified as an important next step. In Malawi, a steering group member mentioned that no changes in policies are necessary for the vertical scale-up. The Quality Management Department could readily implement it without the need for political support from the senior management team, as the Quality Management Department has the mandate to improve DHMTs’ leadership and management.

The majority of country team and national level participants in Uganda and Malawi stated that it remains to be seen whether there will be financial support for integration of (components of) the intervention. In Uganda, for example, it is still unclear whether additional financial resources for the implementation of the QI framework will be secured. In addition, the country team participants and steering group focal person noted that the human resources management capacity of the regional-level QI teams that will be responsible for the implementation of the QI cycles needs to be strengthened to enable them to take on this role. This is in line with what several participants said during the scale-up assessment and country team reflections in Malawi, where over the duration of PERFORM2Scale the country team, resource team and steering group members have not been able to gain the interest of other donors. According to some of the national level and country team participants, this was due to a lack of donor coordination, and to competition between donors and between MoH directorates trying to secure donor funding for their activities. In addition, integration of the intervention into an (existing) policy creates a certain dependency on the functionality and acceptability of existing structures responsible for policy implementation. Functionality and acceptability, however, is not a given in the decentralized context of Malawi, where limited resources and changes in leadership and responsibilities limit the functionality and acceptability of the satellites of the MoH’s Quality Management Department.

“You find that it’s very difficult for them [satellite officers] to do the job because of seniority and also because of some lack of clarity in their mandate. Because, technically, of course it’s debatable what we need the Quality Management Department for.” —DHMT member, Malawi, 2021

In Ghana, national level participants explained that due to a lack of change agents and limited involvement of key decision makers, the majority of national-level stakeholders were unaware of the intervention and its potential scale-up, which resulted in a lack of political and financial support for the vertical scale-up.

#### Windows of opportunity

In Malawi and Uganda, unlike in Ghana, it appeared that a ‘window of opportunity’ enabled vertical scale-up. In Malawi, country team participants explained that the Quality Management Department is a relatively new department in the MoH. They further explained that this created a window of opportunity for the vertical scale-up, as the Quality Management Department was particularly interested in PERFORM2Scale, as it advanced its leadership and management agenda and potentially strengthened its position within the MoH. In Uganda, the country team and steering group focal person explained that they became aware that the five-year QI strategy was set to expire in 2020 and that the MoH was considering a revised supportive supervision strategy. They mentioned that this created a window of opportunity, where the country team and steering group focal person developed the idea of integrating certain unique components of the intervention (the focus on continuous reflection within the action cycles and the focus on workforce performance) that have been absent from the previous QI framework/strategy and its PDSA.

“Having worked with Perform one [pilot project] and two [PERFORM2Scale: on scale-up], we identified that we could also use more or less the similar approach or science to improve performance at management and leadership level, so we thought as we were developing the second QI framework, we would take advantage of that new process and convince stakeholders to include this aspect of human resource performance improvement.” —Steering group, Uganda, 2021

#### Flexible scale-up approach

One of the other facilitators identified in the three countries concerned taking a flexible approach towards scale-up, allowing adaptations to the context, its systems and/or certain blockages and circumstances. In Ghana, several participants at district and national level explained that through continuous dialogue and reflection between the resource team and country team, certain adaptations of the intervention were made to respond to operational challenges and to facilitate the horizontal scale-up. For example, it was decided to include sub-district-level management teams in intervention workshops, to enhance intervention implementation. In Malawi and Uganda, mostly national level participants explained that over time they have adapted their approach to vertical scale-up. In Malawi, the resource team and steering group participants stated that the flexibility of the country team in their approach to scale-up – i.e. being open to adapt the approach based on suggestions from MoH stakeholders – has been very important in the scale-up. This resulted in a scale-up approach that was driven by the Quality Management Department. Country team members in Uganda considered that at the start of the project they had focused too much on preserving the intervention and scale-up strategy as developed by PERFORM2Scale, but that because of resistance from the stakeholders in the technical working groups and discussions with the steering group focal person they realized the importance of adapting the scale-up strategy to the Ugandan context. This resulted in reflections about what exactly the differences were between the PDSA and the action research cycles of the intervention, what niche/possible contribution PERFORM2Scale would make towards quality improvement and how those contributions could be ‘sold’ to the stakeholders as a way to engage them.

“And this is something that we have learned over time: the issue of context is key to scale-up itself. What does it mean? It means you have to listen in to what the context is saying. You have to listen to what is available in the context and then find those windows of opportunities that R1 has been talking about, but at the same time you have to take another moment and reflect and say, ‘Alright, so how can we go about this?’.” —Country team member, Uganda, 2021

This flexibility in approach also included a deliberate shift in language used with the relevant stakeholders. Instead of talking about scaling up the intervention in Uganda, emphasis was put on ‘improving on’ or ‘integrating with’ the QI framework, highlighting that this is not a new intervention, but rather improves existing efforts.

#### Understanding and using the context of decentralization

Another facilitator of scale-up that appeared in two countries was the context of decentralization. The context of decentralization influences the roles and responsibilities of stakeholders involved in the (health) systems and, therefore, influences the politics and power dynamics around scale-up. In Ghana, although the health sector has been decentralized, a strong hierarchy is pervasive. In hindsight, all country team members realized that the composition of the resource team could form a barrier to vertical scale-up, as DHMT members do not have any substantial influence on national-level stakeholders. Country team participants also explained that, in hindsight, targeting the national level for setting up the steering group might have been less suitable in the Ghanaian context. Stronger engagement of the regional level – by setting up a regional scale-up steering group, for example – could have been a more appropriate structure for steering the scale-up. The regional level has substantial influence on the districts and thus on the implementation of the intervention and horizontal scale-up. In addition, through their review meetings, the regional level could have advocated for the intervention with national-level stakeholders to steer vertical scale-up.

Decentralization also determined certain policy priorities and, therefore, influenced the interest in the intervention and its scale-up. In Malawi, scale-up took place in a transition phase of decentralization. Several resource team participants mentioned that the intervention, therefore, fits well, as it enables DHMTs to strengthen capacity and optimize their increased decision-making space. However, the decentralization process in Malawi is dynamic, and this resulted in a lack of clarity around existing structures, roles and responsibilities, and mandate between the MoH and the Ministry of Local Government and Rural Development. Country team participants explained that setting up the steering group (in 2018) was challenging, as it was unclear whether PERFORM2Scale would fall under the MoH or the Ministry of Local Government and Rural Development. In addition, within the MoH, there were discussions about under which department the intervention fits best.

“… We were being pushed from the Ministry of Health to Local Government, and even within the Ministry of Health, it was like… should it be the Planning, should it be the Human Resources and so forth? Even when we are presenting at technical working groups I have been told, ‘We do not need you here. You better liaise with the Ministry of Local Government.’ ... So we have been to Human Resources, we have been to Planning, but to meet the Quality Management Department director was not easy, so that was why we met him much later when we had been told to go to Local Government. But when he got the concept of PERFORM2Scale, he grabbed it by the horns and said it was something he would go for, and that is why today vertical scale-up has gone on very well because it has been accepted, and even other directors are appreciating it.” —Country team member, Malawi, 2021

## Discussion

This comparative case study has shown that scale-up is a challenging and non-linear process. In all three countries we conclude that the horizontal scale-up took place as envisaged during the project in terms of the number of districts adopting the intervention (from three to nine per country). It remains to be seen, however, whether horizontal scale-up will continue beyond the project. The role of the resource team in the horizontal scale-up varied across the countries. In Ghana and Malawi, the lead role in horizontal scale-up moved from the research team to the resource team, whereas in Uganda the research team continued to be in the lead. Furthermore, the vertical scale-up has been quite challenging in all countries, partly because of challenges in the functioning of the steering groups. In Ghana, a strategy for vertical scale-up remained absent. In Malawi and Uganda, a strategy has been developed, but at the time of data collection it was unknown whether the identified strategies will succeed, as they depend on, among others, the allocation of (extra) resources, the revival of existing structures, and enhanced capacity of people who will be involved. Without vertical scale-up, the continuation of the horizontal scale-up is likely to be seriously constrained.

This study identified several key factors and processes of scaling up. These facilitators are strongly interlinked. The identified facilitators in this study go a step further than the identification of systemic and general contextual factors influencing scale-up, such as the need for aligning the scale-up with policies and guidelines and the importance of political will, as often discussed in the literature [13]. Our findings provide insights into factors and processes that positively facilitated scale-up within contexts shaped by different systemic and general contextual factors. Having a shared vision about the scale-up and a scale-up strategy are critical, as was also identified in the WHO/ExpandNet scale-up guidance documents [4]. This study emphasizes the importance of a collaborative approach among relevant stakeholders in the development of a scale-up strategy so that there is a common understanding and vision concerning how and what to scale up in a specific context. The importance of change agents who create buy-in from different key decision makers shows that scale-up is strongly influenced by stakeholders’ interests and incentives, as well as by power dynamics and politics. For example, in Ghana, although the health sector has been decentralized, there is still a clear hierarchy across the different levels [15], which thus needs to be taken into account during scale-up. In Malawi, recent changes as a result of decentralization sometimes cause friction and a lack of clarity regarding the different roles and responsibilities between the MoH and the Ministry of Local Government and Rural Development [11]. The importance of power dynamics between different stakeholders involved in scale-up, therefore, requires thinking and working politically [16]. A window of opportunity can trigger scale-up, as illustrated in Malawi and Uganda, and shows the importance of identifying and seizing the opportunity. Lee, Van Nassau [17] also indicate that scale-up is not linear and depends on an interplay of factors that together might or might not create a window of opportunity for scale-up. This study also underlines the importance of a flexible approach towards scale-up. In the literature, considerable attention is paid to adaptation of the intervention, to ensure it fits across different contexts [18]. Besides the importance of adaptation of the intervention that is being scaled up, this study also highlights the importance of flexibility in terms of valuing and acting on input into the scale-up strategy from relevant stakeholders. This contributes to a feeling of ownership of the scale-up and increases the chances of political and financial support from, for example, national institutions.

The study findings show that full integration of the intervention into the health system was challenging in all countries. Woltering and colleagues [19] have also observed that “dealing with or influencing” the existing system is difficult and seldom leads to successful vertical scale-up. PERFORM2Scale took spreading the intervention (horizontal scale-up) as the starting point and assumed that embedding the intervention into the system in which (vertical) scale-up was to take place would increase the chances for success [20]. However, Woltering, Fehlenberg [19] state that projects should focus on bringing sustainable systems change at scale, rather than aiming for horizontal scale-up and then embedding the intervention in the system.

If the scale-up of the management strengthening intervention in PERFORM2Scale had been approached from a systems change perspective, the scale-up approach would have looked different and might have had different outcomes. Instead of focusing on scaling up the specific management strengthening intervention, which was the aim of the research project and the request for proposal, the focus could rather have been on how to transform the current normal to a ‘new normal’, as aligned with the transition theory [21–24]. Instead of having the ‘solution’ as the starting point (in this case, the management strengthening intervention), the problem could have been the starting point (in this case, DHMTs making limited use of decision space, leading to suboptimal health service delivery and workforce performance). Based on the understanding of this problem, a combination of several interventions or approaches could have been developed and implemented at the same time, through a collaborative approach with a broad variety of stakeholders from different disciplines and backgrounds (Rotmans and Loorbach [25] call this ‘transition experiments’). Depending on the country, one could think about the integration of a management course into the public health curriculum, strengthening the regional level in providing technical assistance to DHMTs or supporting district councils in local resource allocation, and strengthening management capacity of DHMTs on the job through action research and inter-district learning meetings, as was done in PERFORM2Scale. When several interventions are implemented, it is important to explore which intervention or combination of interventions is able to succeed in contributing to a ‘new normal’. Cyclical and participatory approaches to implementation of the interventions, with a focus on (social) learning are, therefore, crucial. Based on the learnings, the interventions can be adapted to allow for improvement [21, 23–26].

The findings of this study show that it is challenging to first focus on the spread an intervention, and then on its integration in the existing system. A systems change perspective encourages reflection on how to “create a new normal” and sometimes even change (elements) of the existing system from the start. This could be best done through flexible and adaptive approaches while implementing a mix of interventions during a longer time period. Though approaching scale-up from a systems change perspective sounds promising, there are as yet few examples; therefore, limited evidence is available to showcase the effectiveness [19]. This could be because of the challenging context, particularly in low- and middle-income countries. In addition, the donor landscape seldom allows for flexible and adaptive approaches with a mix of interventions over long time periods. While this is the case, our discussion shows that a systems change approach offers relevant lessons for scaling up interventions in complex systems.

A strength of this study is the use of a comparative case study approach, which allowed to study, compare and follow the scale-up of the intervention in different contexts over time, using a variety of data collection methods and involving a variety of stakeholders. During the second round of data collection, the interviews in Ghana and Uganda had to be conducted online because of the COVID-19 pandemic, which may have resulted in less rich data in Ghana and Uganda, compared to Malawi. In Uganda, the first country team reflection did not take place. This may have resulted in less deeper reflection of the findings at that time. However, the data collected during the first round were validated by the country teams and discussed during the second round of country team reflections.

## Conclusion

The experience of scaling up a district health management strengthening intervention in Ghana, Malawi and Uganda has revealed several interlinked facilitators of scale-up. When scaling up, it is important to take the system in which the intervention is being scaled into account, but it is questionable whether this is always sufficient for scale-up to succeed. Looking at scale-up from a systems change perspective provides relevant lessons for scaling up future health interventions, which is essential for achieving Universal Health Coverage. 

## Supplementary Information

**Additional file 1: Supplementary file 1. **Overview of differentstructures of Ghana, Malawi, Uganda.

## Data Availability

The data that support the findings of this study are available from the corresponding author, Susan E. Bulthuis, upon reasonable request.

## Abbreviations

DHMT: District Health Management Team
MoH: Ministry of Health
PDSA: Plan, Do, Study, Act
QI: Quality Improvement

## Footnotes

1 Researchers from the School of Public Health (Ghana), Reach Trust Malawi (Malawi) and Makerere University School of Public Health (Uganda).

2 https://www.perform2scale.org/management-strengthening-intervention-toolkit/introduction-to-the-msi
