# Supplementary figures and images for: How to scale-up: a comparative case study of scaling up a district health management strengthening intervention in Ghana, Malawi and Uganda
Source: BMC Health Serv Res. 2023 Jan 16;23:35. doi: 10.1186/s12913-023-09034-1 (PMC9840942; doi:10.1186/s12913-023-09034-1)

**Supplementary file 1**

*
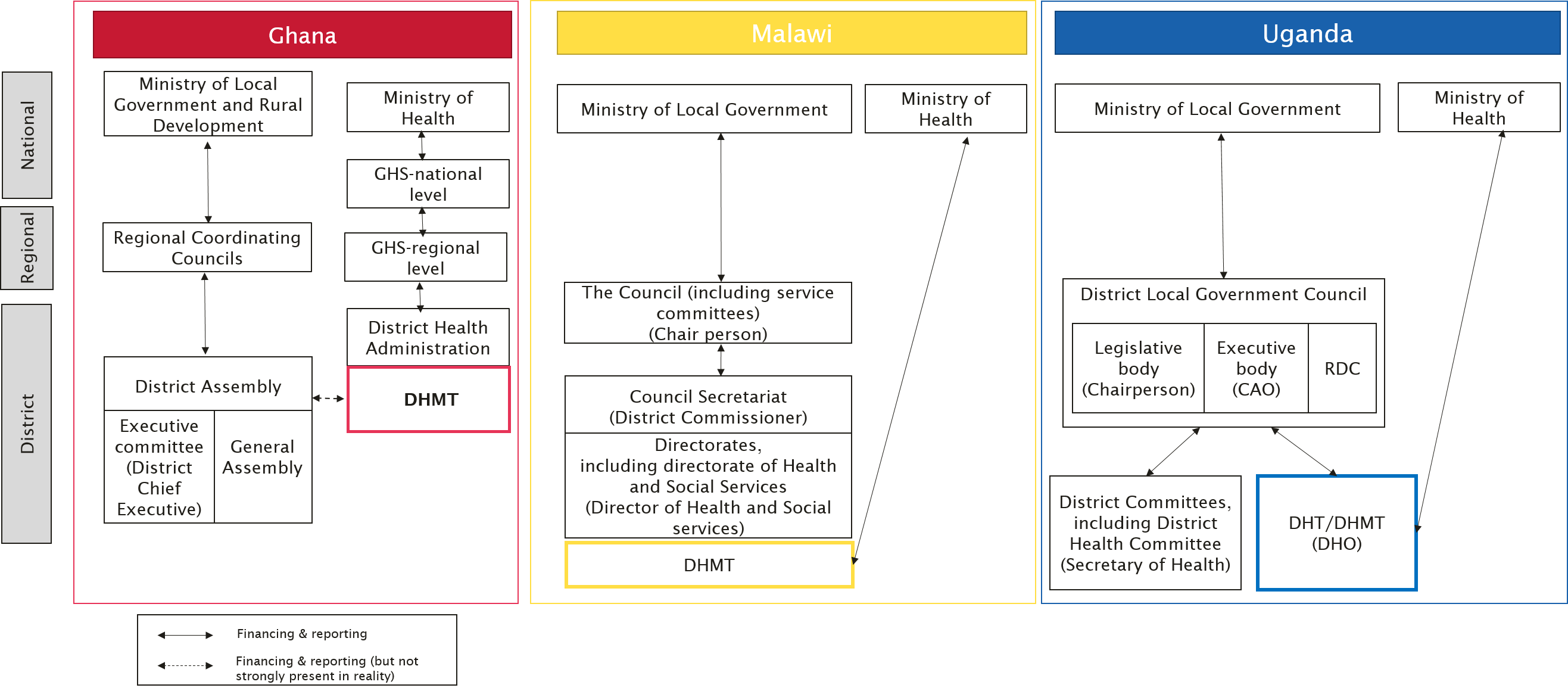
*

*Overview of different structures of Ghana, Malawi, Uganda.*

Supplement: Supplementary file 1 — Additional file 1: Supplementary file 1. Overview of differentstructures of Ghana, Malawi, Uganda. [file 12913_2023_9034_MOESM1_ESM.docx]
